# Network based stratification of major cancers by integrating somatic mutation and gene expression data

**DOI:** 10.1371/journal.pone.0177662

**Published:** 2017-05-16

**Authors:** Zongzhen He, Junying Zhang, Xiguo Yuan, Zhaowen Liu, Baobao Liu, Shouheng Tuo, Yajun Liu

**Affiliations:** School of Computer Science and Technology, Xidian University, Xi’an, PR China; Instituto Nacional de Medicina Genomica, MEXICO

## Abstract

The stratification of cancer into subtypes that are significantly associated with clinical outcomes is beneficial for targeted prognosis and treatment. In this study, we integrated somatic mutation and gene expression data to identify clusters of patients. In contrast to previous studies, we constructed cancer-type-specific significant co-expression networks (SCNs) rather than using a fixed gene network across all cancers, such as the network-based stratification (NBS) method, which ignores cancer heterogeneity. For each type of cancer, the gene expression data were used to construct the SCN network, while the gene somatic mutation data were mapped onto the network, propagated, and used for further clustering. For the clustering, we adopted an improved network-regularized non-negative matrix factorization (netNMF) (netNMF_HC) for a more precise classification. We applied our method to various datasets, including ovarian cancer (OV), lung adenocarcinoma (LUAD) and uterine corpus endometrial carcinoma (UCEC) cohorts derived from the TCGA (The Cancer Genome Atlas) project. Based on the results, we evaluated the performance of our method to identify survival-relevant subtypes and further compared it to the NBS method, which adopts priori networks and netNMF algorithm. The proposed algorithm outperformed the NBS method in identifying informative cancer subtypes that were significantly associated with clinical outcomes in most cancer types we studied. In particular, our method identified survival-associated UCEC subtypes that were not identified by the NBS method. Our analysis indicated valid subtyping of patient could be applied by mutation data with cancer-type-specific SCNs and netNMF_HC for individual cancers because of specific cancer co-expression patterns and more precise clustering.

## Introduction

Cancer is a heterogeneous disease which is formed by various subtypes. In an organ, different tumour subtypes is a reflection of certain molecular oncogenic processes and different clinical outcomes, which means these subtypes are supposed be regarded as different kinds of cancers in treatment design [1]. As cancer genomic, transcriptomic and epigenomic information is becoming increasingly available, the stratification of tumours into valid clinic subtypes according to molecular data is crucial for guiding better treatment and prognosis. Due to the growth of mass high-throughput omics data, standard unsupervised clustering methods are used to cluster samples [2–4], such as non-negative matrix factorization (NMF) and hierarchical clustering. Computational methods can be used to identify tumour subtypes which have different survival rates, tumour levels or stage, histological types, and responses of treatment.

Key point of previous studies is utilizing messenger RNA (mRNA) expression data [2–3,5] to successfully group patients based on similarities in gene expression into clinically relevant phenotypes. While somatic mutations can make mutated genes lose function and offer more clinical guidance [6–8], these mutations are not universal phenomenon among patients [9–10]; thus, it is impossible to directly measure the similarity among tumours using mutated genes. The most advanced state-of-the-art integrative method for cancer analysis, network-based stratification (NBS), considers the sparsity of mutations by searching for mutational consistencies at the network level rather than at the individual gene level. This method can be used to identify patients’ subgroups with similar molecular-network patterns by propagating mutation labels on a prior gene interaction network.

As the molecular network can be regarded as an obvious sign for interactions and relationships between molecules, it is adopted for the biological discovery of complex diseases [11–12]. NBS and current studies based on NBS all utilized the same prior networks across cancers [13–16]. However, we recognize that the regulation of gene expression levels among genes might be related to the type of cancer, and different cancers correspond to different regulations. Yang Y et al. constructed 4 cancer-type-specific co-expression networks (CNs) and revealed that, the hub genes which can be found in specific cancer networks are merely slightly overlap [17]. Thus, for a distinct cancer, the mutations should be mapped onto a network that was derived specifically from that cancer. A CN is constructed based on the correlations among the quantitative gene expression levels in tumours, and the CN is presented as a graph in which the nodes correspond to genes, and the edges correspond to co-expressions among the genes. A CN contains more precise information regarding the connectivity among genes in individual cancer types than prior networks. CNs have been shown to be helpful for describing the pair wise relationships among gene transcripts [18–19]. Genes with similar or correlated expression patterns might contribute to the same regulatory function, and gene co-expression patterns in a CN may lead to more insightful discoveries regarding the underlying regulatory mechanisms [20–21].

Thus, we propose a method based on NBS for the stratification of cancer by constructing a gene network for each cancer. In this study, we integrated somatic mutation data and gene expression data. For each cancer, the gene expression data were used to construct a CN, and the somatic mutations in the genes were mapped into the network and propagated, which was useful for further clustering.

Furthermore, network-regularized NMF (netNMF) clustering using the NBS method has been shown to be better than the standard NMF method. netNMF first maps the samples with smoothed mutations into a lower k-dimensional feature vector space using netNMF matrix factorization, which constrains the genes with respect to the gene interaction network; then, the sample category is determined by the column with the largest value among the k feature vectors. This clustering is reasonable because it is possible to select the class that has the largest weight concerning the most relevant feature vector [7], but the class cannot be easily estimated if there are two similarly large values. The class of the samples may be more precisely identified by clustering the factorized low rank feature vectors of the samples using a clustering method, such as hierarchical clustering.

**Fig 1:**
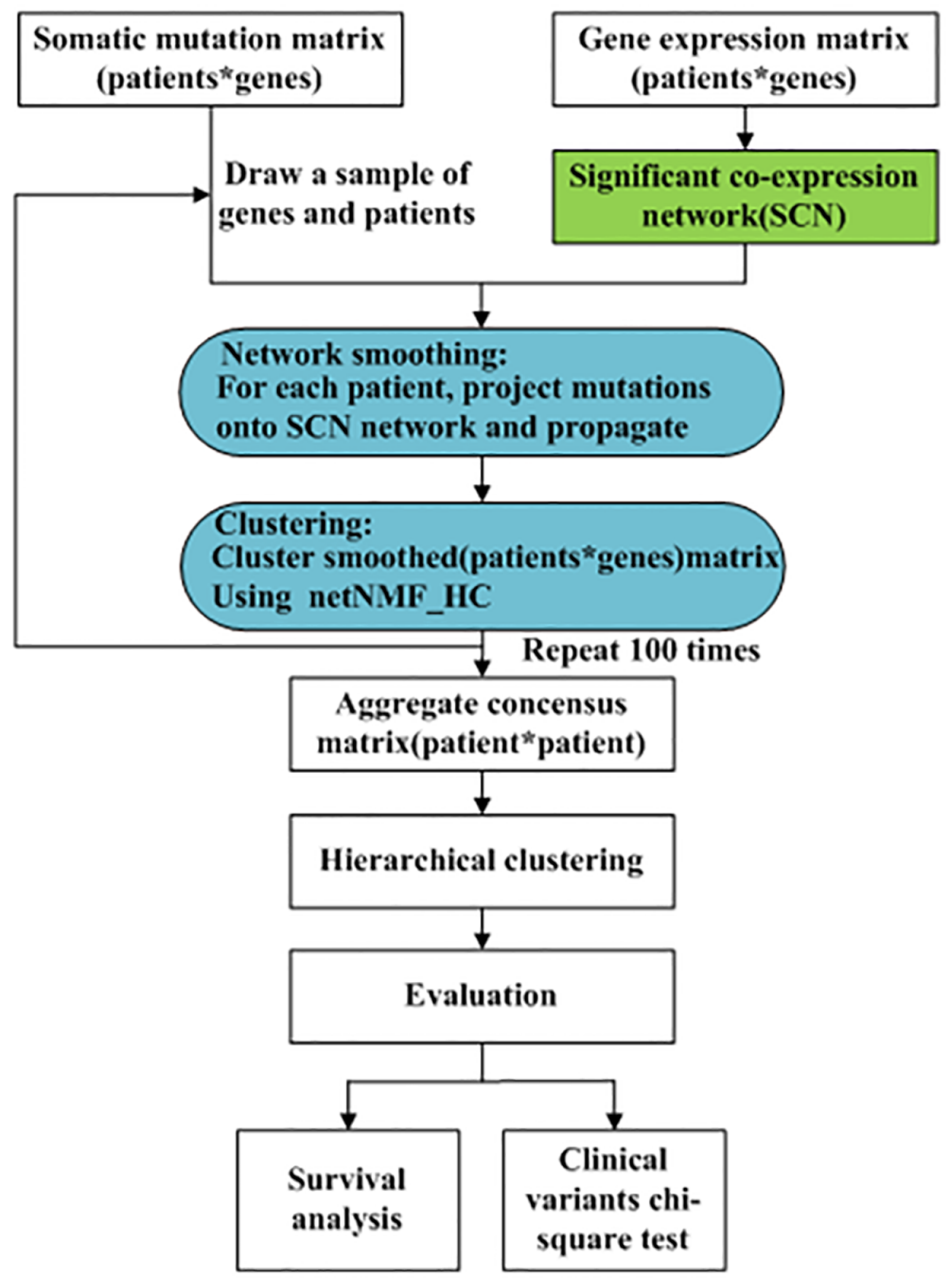
Workflow of our method.

In this study, we propose an improved stratification method based on NBS through combining gene expression data and somatic mutation. First, we constructed a significant co-expression network (SCN) using gene expression profiles for each cancer type. Then, for patients, we projected the profile of mutation onto the cancer-type-specific SCN network. Network propagation was applied to diffuse the effect of mutation over its network neighbourhood. Finally, the matrix of the ‘network-smoothed’ was stratified into different subtypes with numbers ranging from 2 to 8 via the netNMF_HC clustering method. The effectiveness of our method was evaluated based on the relevance of our subtypes and clinical outcomes and compared with NBS, which used prior gene networks and the netNMF clustering method. The results showed that our method outperformed NBS for the three cancers and identified the survival-relevant subtypes of uterine endometrial carcinoma (UCEC), which were not identified by NBS. The workflow of our method is described in Fig 1.

## Materials and methods

### Data and pre-processing

#### TCGA somatic mutation data and mRNA expression data

For comparing with NBS fairly, the somatic mutation profiles for serous ovarian cancer (OV), UCEC, and lung adenocarcinoma (LUAD) from TCGA and two prior gene-gene interaction networks STRING and Humannet were collected from materials of Hofree et al [4]. The RNAseqv2 gene expression profiles for the three cancers were downloaded from the Synapse database (https://www.synapse.org/#!Synapse:syn300013) in which the expression levels are normalized using MAS5. The expression data were used to construct an SCN for further analysis. As shown in Table 1, the ovarian carcinoma dataset contained somatic mutations in 9,850 genes from 356 samples and gene expression profiles for 20,534 genes from 430 samples. The LUAD dataset contained somatic mutations in 15,967 genes from 381samples and gene expression profiles for 20,199 genes from 576 samples. The UCEC dataset contained somatic mutations in 17,968 genes from 248 samples and gene expression profiles for 20,531 genes from 381 samples.

**Table 1:** Sample sizes, somatic mutations and gene expression profiles for three cancers.

	Somatic mutation data		Gene expression profiles	
	Sample size	Number of genes	Sample size	Number of genes
OV	356	9,850	430	20,534
LUAD	381	15,967	576	20,199
UCEC	248	17,968	381	20,531

First, the mutation matrix *F* was generated based on samples with somatic mutations. *F* is binary as follows: if any gene mutates (a single nucleotide base change or an insertion or deletion of bases) in a certain sample relative to the germ line, the mutation is marked the number 1; otherwise, 0 is assigned. The expression matrix *E* is a real matrix, and each of its entries indicates a normalized given gene expression in a given sample. In all matrices, the samples and genes are represented by the rows and columns. We filtered samples with fewer than 10 mutations in mutation matrix *F* and genes with 0 expression in all samples in the gene expression matrix *E*. The clinical data, including survival, stage and grade of the three cancers, were gotten from the Synapse database (https://www.synapse.org/#!Synapse:syn300013) and were applied in evaluation of the relevance of the identified subtypes and clinical outcomes.

#### Gene interaction networks

The patient mutations were projected onto the gene interaction networks as follows: NBS utilizes STRING v.9 [22] and HumanNet v.1 [23], and our method employs an SCN that was constructed by gene expression profiles.

### Method

#### Construction of the SCN

For each cancer type, an SCN was constructed to represent the significant correlations between a pair of genes without a prior interaction network. First, the absolute Spearman’s rank correlations and the corresponding p-values of these correlations were calculated for all gene pairs based on the expression profile matrix of each cancer type. The original co-expression network (CN) was constructed based on the absolute correlations as the weight of the edges. Then, the expression of gene pairs was considered significantly correlated if the q-value (Bonferroni corrected p-value) of their correlation was smaller than 0.05. The SCN was obtained by filtering the edges with correlations whose corresponding q-values were greater than 0.05. Thus, the SCN is an unweighted undirected network in which each vertex denotes a gene used in our work. Spearman’s rank correlation was chosen as a measure to evaluate the relevance of two genes because it can detect nonlinear relationships, and it has been verified to have better performance than other measures, such as Euclidean distances and Pearson’s correlations, in measuring the similarity between genes [24]. Spearman’s rank correlations and the corresponding p-values were calculated using the function corr() in MATLAB.

Finally, three cancer-type-specific SCNs were obtained, in which two genes are connected if they are estimated to be significantly co-expressed.

#### Network smoothing

For each patient of each cancer, we mapped the mutation profile onto the constructed cancer-type-specific SCN. Network propagation [25] was used to propagate the mutation signal among networks. The key is to spread the mutation information of every gene to its neighbours iteratively until a stable state is achieved. The algorithm used is as follows:

**Step1:** Construct the degree-normalized matrix *W*′ = *D*^-1/2^*WD*^-1/2^, where *D* is a diagonal matrix, and its columns sums *W* on the diagonal; *W* is the adjacency matrix of the SCN network; and the diagonal elements of *W* are set to zero. The normalized matrix *W*′ is utilized for the following smooth process.
**Step2:** Iterate *F*_*t*+1_ = *αF*_*t*_*W*′+ (1−*α*)*F*_0_ until convergence is achieved (the matrix norm of *F*_*t*+1_ −*F*_*t*_ <1×10^−7^), where *F*_0_ is the patient-by-gene somatic mutation matrix, and the parameter *α* is a tuning parameter in (0, 1) that governs the relative amount of the information from the gene's neighbours and its initial mutation information. It should be noted that self-reinforcement should be avoided because the diagonal elements of the adjacency matrix *W* are set to zero at the beginning. The optimal value of *α* is set as 0.7and is represented in NBS [4]. Each row in *F*_*t*_ represents the smoothed mutation of genes in a sample after it is influenced by its neighbours.
**Step3:** For *F*_*t*_, quantile normalization is regarded as the guarantee for patient to follow the same smoothed mutation profile distribution. *F* was applied to show the transpose of the final normalized and smoothed mutation matrix.

#### Improved netNMF_HC

NMF is a matrix factorization algorithm which can resolve a matrix into two lower rank non-negative matrices [26]. netNMF can be regarded as an evolution of NMF which rules NMF to keep the gene interaction network structure. The objective is producing two non-negative matrices, *W* and *H*, to minimize the following function using an iterative method [27]:

$$\underset{W,H>0}{\text{min}}{\left\|F-WH\right\|}_{F}^{2}+\lambda \text{trace}(W^{t}LW)$$ (1)

Where ${\left\|.\right\|}_{F}^{2}$ denotes the matrix Frobenius norm, *W* and *H* are decompositions of the smoothed m by n matrix *F*. *W* is an m by k basis matrix or "metagenes", and *H* is a k by n coefficient matrix. The reduced dimension is controlled by the value k and k is set 2~8.

*L* is the graph Laplacian of the *p*-nearest-neighbour network derived from the original weighted gene co-expression network (CN). If *v*_*i*_ and *v*_*j*_ are two connected vertices in the original network, the weight of edge *w*_*ij*_ of the *p*-nearest neighbour network *w* is as follows:

$$w_{ij}=\{\begin{array}{ll} 1, & \text{if }v_{i}\in N_{p}(v_{j})\;or\;v_{j}\in N_{p}(v_{i})\\ 0, & \text{otherwise} \end{array}$$ (2)

where *N*_*p*_(*v*_*i*_) indicates the set of *p*-nearest neighbours of *v*_*i*_. The graph Laplacian of *w* is *L* = *D*(*w*) − *w*, where *D* is a diagonal matrix with the sums of a column (or row as *w* is symmetrical) of *w D*_*ii*_ = Σ_*j*_*w*_*ij*_ on the diagonal line. We set the number of nearest neighbours *p* = 11, and the regularization parameter *λ* was set to 200, which are on the same scale in NBS [4]. The function was run iteratively until it converged (||*F*–*WH*||<1×10^−3^).

Finally, in previous works, the class of the samples was obtained from the coefficient matrix *H*. *H* is a k by n coefficient matrix, indicating n samples with k feature vectors. For a sample *n*, class $c_{n}=\text{arg}\underset{k}{\text{max}}(H_{kn})$; therefore, the sample belongs to the column number of the feature vector with the largest value.

It is not precise enough to use the netNMF method for determining the category of n samples; for example, if two vector values are almost the same, it may be difficult to determine the final class. Therefore, we propose the improved method netNMF_HC, which considers the k by n coefficient matrix *H* from netNMF to be a low dimension feature space of the patients, and then, *H* is utilized to group samples by hierarchical clustering (HC) to obtain the class of the patients.

#### Consensus clustering

Robust clustering was achieved by applying consensus clustering [28] to produce the final subtypes. Precisely, 80% of the patients and genes were sampled randomly for network smoothing and netNMF_HC was used to perform the clustering. The process was repeated 100 times. The results of the 100 clustering made up the patient-patient similarity matrix. The matrix recorded the frequency of the sampling of each pair of patients and the rates at which the pairs were clustered in same group among all replicates. According to the similarity matrix, hierarchical clustering with average linkage can be produced.

#### Clinical analysis

The analysis of survival was generated by the R “survival” package. Kaplan-Meier survival curves of subtypes and log-rank test p-values were utilized to evaluate the association between the subtypes and patient 10 year survival time. Pearson’s chi-squared test was aimed to evaluate the relationship between the subtypes and tumour grade or stage.

#### Identification of differentially mutated genes in subgroups

The significantly mutated genes of each subtype relative to the remaining subtypes were identified using the significance analysis of microarrays (SAM) method [29] with the network-smoothed mutation data. The q-values were estimated by SAM with the Wilcoxon-rank statistic and 1,000 permutations. Then, the differentially mutated genes with q-values<0.05 for each subtype were selected for further analysis.

We performed a biological processes and pathway enrichment analysis for the significantly differentially mutated genes in each subtype using DAVID 6.8 (https://david.ncifcrf.gov/). Only enriched annotation terms whose q-values were lower than 0.05 were retained.

## Results

We tested our method and the NBS method in ovarian, uterine and lung cancer. In our method, the somatic mutations in these cancer types were propagated onto an SCN, and then, the smoothed mutation profiles were clustered with consensus clustering based on netNMF_HC. In NBS, the somatic mutations in these cancer types were mapped onto the gene interaction networks STRING and Humannet, and then, the smoothed mutation profiles were clustered with consensus clustering based on netNMF. To determine whether the SCN network or the improved clustering method netNMF_HC contribute to our method, we also performed experiments in which only changing the network or the clustering method. The clustering outcomes (cluster number k = 2~8) for each kind of cancer are shown in the S1 File. Finally, different outcomes were observed for a given cancer type and a number of clusters k.

### Clinical analysis

**Fig 2:**
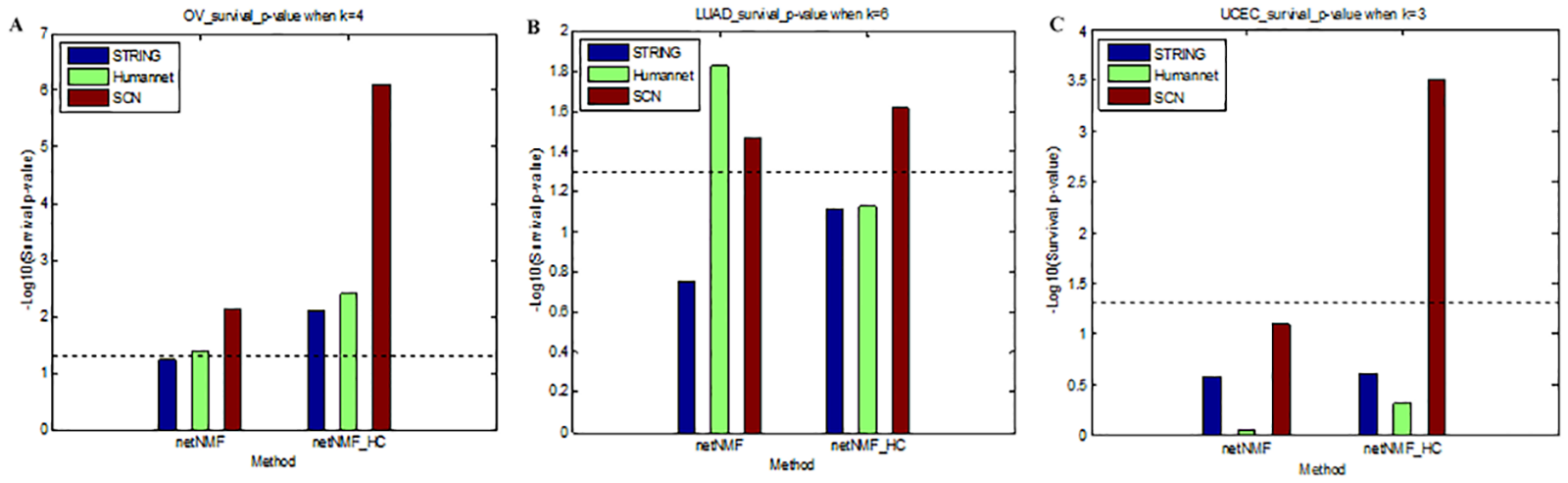
Survival p-values in three cancers with distinct methods. (A) Significance with -Log10(p-value) association between 10 year survival and subtypes obtained by distinct combination of networks (STRING(blue), Humannet(green) and Significant co-expression network(SCN)(red)) and clustering methods (netNMF and netNMF_HC) for ovarian cancer (OV) with cluster number k = 4. (B) for lung cancer(LUAD) with cluster number k = 6. (C) for uterine cancer(UCEC) with cluster number k = 3. Dashed lines represent the -log10(P = 0.05) threshold.

**Fig 3:**
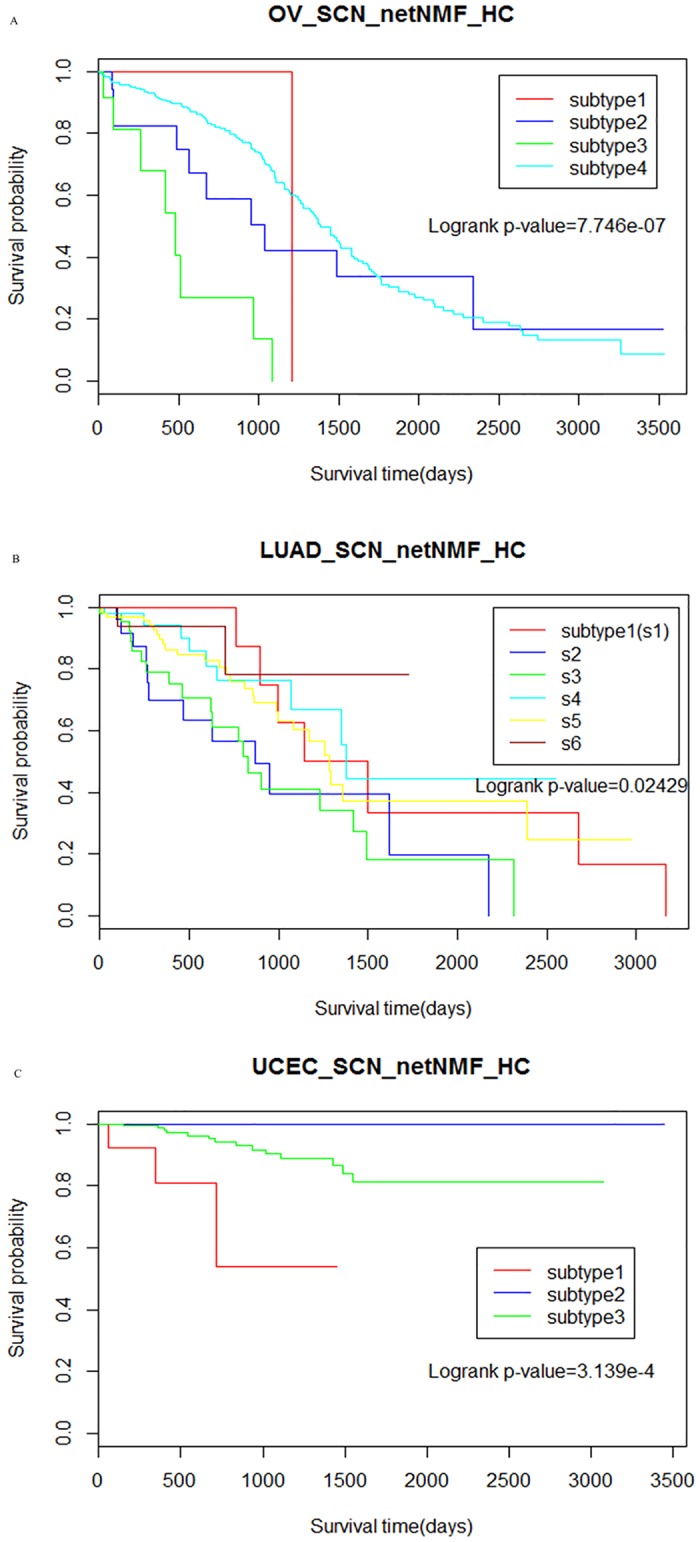
Survival curves of three cancers with our method. (A) Kaplan-Meier survival plots of subtypes obtained by our method that combining the significant co-expression network(SCN) and clustering method netNMF_HC for ovarian cancer (OV) with cluster number k = 4. (B) for lung cancer(LUAD) with cluster number k = 6. (C) for uterine cancer(UCEC) with cluster number k = 3.

The relationship between subtypes and 10 year survival was investigated in the first time. All three cancer subtypes derived from our method were significantly associated with survival in certain cluster numbers (log-rank test p-value < 0.05) as shown in Table 2 and Fig 2. Compared with NBS, when only the network was changed, the cancer-specific SCN performed better than prior network STRING in discovering clinically relevant subtypes for OV and LUAD; when only changing the clustering method, the improved clustering method netNMF_HC performed better than netNMF for OV. When both were changed simultaneously, our method performed better than the NBS method for all three cancers. Additionally, using our method (SCN + netNMF_HC), the OV, LUAD and UCEC samples were divided into 4, 6 and 3 clusters respectively having most significant association with the survival time, each cluster was independent and differed in survival (Fig 3), while the NBS method using the STRING network was less effective (p-values were not significant). Especially, the survival-relevant UCEC subtypes could not be obtained with NBS based on the STRING or Humannet network, which is consistent with a previous work [4]. We then measured the relationship between the subtypes and the tumour grade or stage. Among three kinds of cancer, there was no relationship between the clusters and the tumour grade or stage, except for UCEC.

**Table 2:** Critical relationship between subtypes and survival in 3 tumour types using NBS and improved methods.

Survival p-value	Cluster number k	NBS		Only changing network	Only changing clustering method		Our method
		STRING +netNMF	Humannet +netNMF	SCN+ netNMF	STRING +netNMF_HC	Humannet +netNMF_HC	SCN+NetNMF_HC
**OV**	4	0.058222	0.040828	0.007334	0.00792	0.003891	7.70E-07
**LUAD**	6	0.177624	0.014901	0.033739	0.076693	0.07448	0.024237
**UCEC**	3	0.260764	0.913596	0.080808	0.248289	0.491311	0.000314

Overall, the different networks applied in the work influenced the stratification results. The SCN network performed better than the prior networks STRING and Humannet in most of the studied cancer types. Although the clustering method improvement did not contribute much, the combination of SCN network and netNMF_HC clustering method achieved better performance than NBS for all three cancers.

### Identifying differentially mutated genes in subgroups

**Fig 4:**
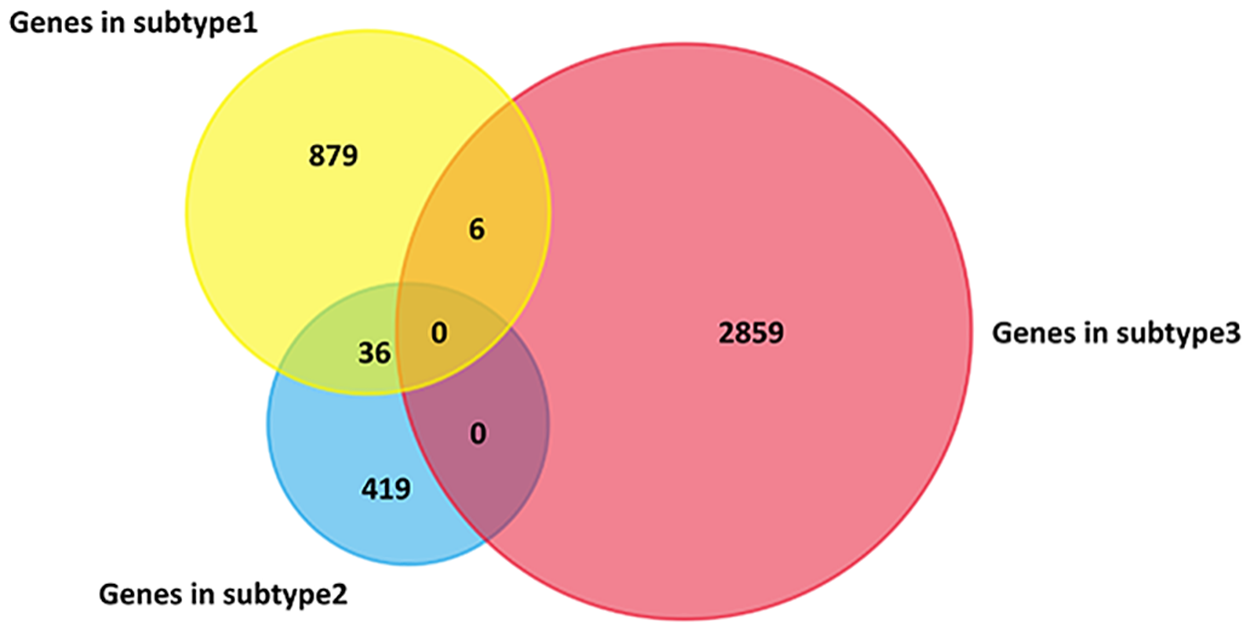
Overlap of genes distinguishing the three subtypes of UCEC.

**Fig 5:**
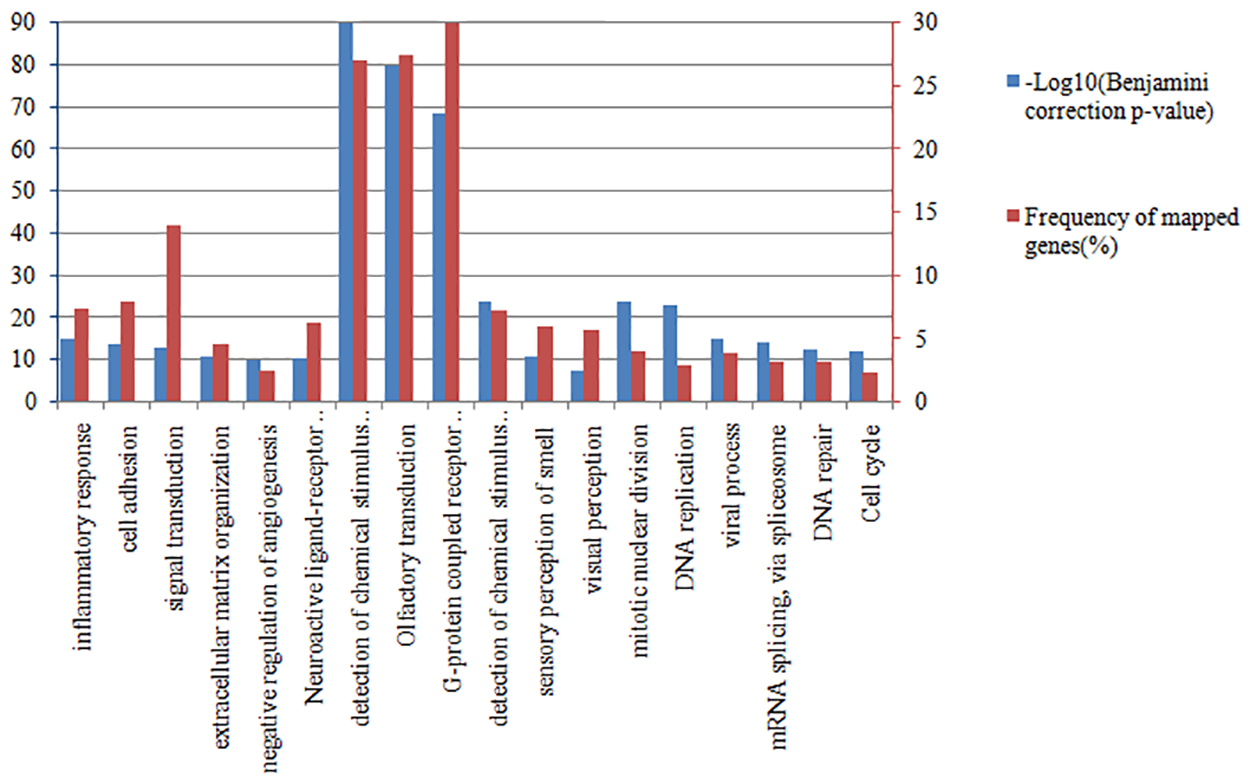
Top enriched biological processes and pathways of the differentially mutated genes in each subtype of UCEC. Bars indicate the significance with -log10(Benjamini correction p-value) (blue) and the frequency of the mapped genes (red) of the corresponding function.

We further identified the significantly differentially mutated genes in each subtype of UCEC for instance. The overlap of these gene sets is very few, which means these genes are specific to certain subtype (Fig 4). In addition, the enriched biological processes and pathways of these genes are also distinct for different subtypes (Fig 5).

### Mutation pattern analysis

**Fig 6:**
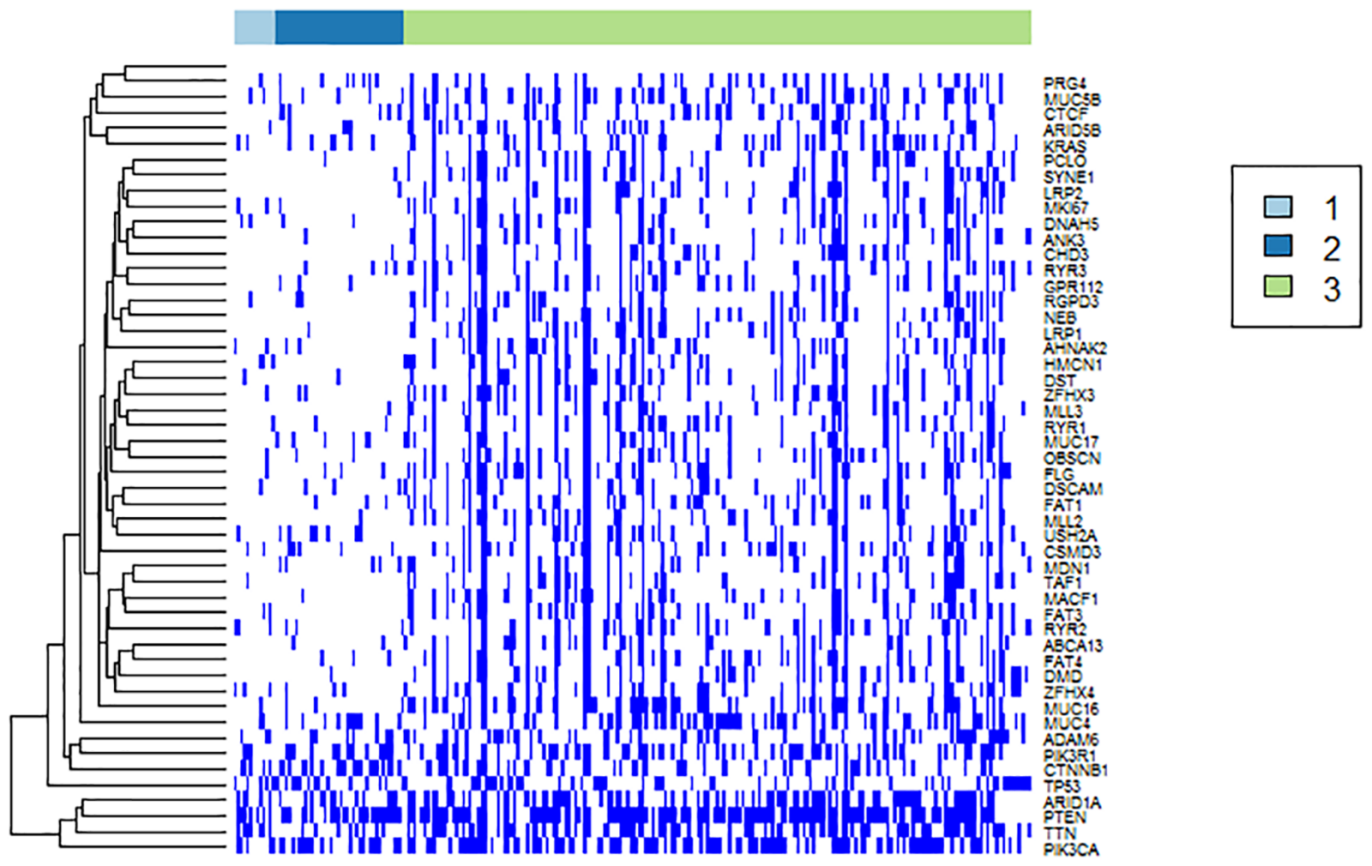
Somatic mutation patterns in three UCEC subtypes. Each row indicates a highly frequent gene, and each column denotes a sample. Dark colours in the figure indicate that a mutation occurred in a gene in a sample.

As shown in Fig 3C, three UCEC subtypes were obtained. Cluster 1 (red) has the worst survival and cluster 2 (blue) has the best survival. The mutation patterns (before the network smoothing) of the three UCEC subtypes which are predictive of survival was analysed. As shown in Fig 6, the three subtypes have different mutation schemas, and cluster 3 harboured more mutations than the other clusters. Both *PIK3CA* and *PTEN* alterations have been reported to have strong relationships with UCEC [30].

## Discussion

Exome and whole genome sequencing have provided a large amount of genomic and transcriptome data. These data enable the stratification of patients into clinically relevant subtypes, making molecular-driven diagnoses and therapy feasible. Network-based methods have integrated mutations and prior gene interaction networks to identify the clinically relevant subtypes. In this study, we showed that due to tumour heterogeneity, combination of a cancer-type-specific SCN and improved clustering method for each cancer type can achieve a superior stratification compared to using the prior fixed gene network for all cancers and often also has a better predictive performance of survival. This finding indicated that the cancer-type-specific gene SCNs can offer useful individual cancer biological knowledge for effective subtyping.

Clinically relevant tumour subtypes of some cancer types may be driven by various mechanisms, such as copy number aberration or methylation, besides somatic mutations and gene expression levels. Integrating multiple types of molecular data to discover truly predictive subtypes is essential for the future. Based on our analysis, the top 50 genes that are mutated frequently across tumour samples (generally called mutation drivers) can distinguish the UCEC subtypes by the mutation patterns. Driver genes may be beneficial for stratification because they are signatures that can capture the differences among the subtypes. In conclusion, clinically relevant subtyping performance may be further improved by integrating clinical driver genes obtained from integrated molecular data and cancer-specific networks.

## Supporting information

S1 File Significant p-value of association between subtypes and survival for three cancers. (PDF)
