# Supplementary material for: Network based stratification of major cancers by integrating somatic mutation and gene expression data
Source: PLoS One. 2017 May 16;12(5):e0177662. doi: 10.1371/journal.pone.0177662 (PMC5433734; doi:10.1371/journal.pone.0177662)
Supplement: S1 File — (PDF) [file pone.0177662.s001.pdf]

S1 File. Significant p value of association between subtypes and survival for three cancers.

Table s1. Significant p-value of association between subtypes and survival for OV.

Survival p-value	NBS		Only changing network	Only changing clustering method		Our method
	STRING +netNMF	Humannet +netNMF	SCN +netNMF	STRING +netNMF_HC	Humannet +netNMF_HC	SCN +netNMF_HC
2	0.014115	0.034567	0.03181	0.037	0.034567	0.03181
3	0.026325	0.01221	0.026908	0.011027	0.015432	0.026908
**4**	**0.058222**	**0.040828**	**0.007334**	**0.00792**	**0.003891**	**7.70E-07**
5	0.047173	0.001697	0.034637	0.043855	0.005563	1.13E-07
6	0.034923	0.000715	0.009672	0.036394	0.000235	7.90E-07
7	0.033383	0.001651	0.010721	0.010927	0.000466	1.63E-06
8	0.098677	0.002417	0.098409	0.021038	0.001389	9.60E-07

Table s2. Significant p-value of association between subtypes and survival for LUAD.

Survival p-value	NBS		Only changing network	Only changing clustering method		Our method
	STRING +netNMF	Humannet +netNMF	SCN +netNMF	STRING +netNMF_HC	Humannet +netNMF_HC	SCN +netNMF_HC
2	0.098955	0.283948	0.168339	0.969774	0.283948	0.107694
3	0.284578	0.207168	0.027131	0.273556	0.06051	0.03159
4	0.031259	0.110288	0.044299	0.236736	0.011364	0.04235
5	0.115976	0.491195	0.244748	0.037946	0.033581	0.039724
**6**	**0.177624**	**0.014901**	**0.033739**	**0.076693**	**0.07448**	**0.024237**
7	0.169893	0.14773	0.04622	2.33E-06	0.07249	0.029206
8	0.049142	0.077195	0.062715	0.016001	0.177267	0.141487

Table s3. Significant p-value of association between subtypes and survival for UCEC.

Survival p-value	NBS		Only changing network	Only changing clustering method		Our method
	STRING +netNMF	Humannet +netNMF	SCN +netNMF	STRING +netNMF_HC	Humannet +netNMF_HC	SCN +netNMF_HC
2	0.471122	0.334912	0.252608	0.232319	0.232319	0.252608
**3**	**0.260764**	**0.913596**	**0.080808**	**0.248289**	**0.491311**	**0.000314**
4	0.821372	0.426871	0.181596	0.52218	0.552503	0.05898
5	0.685519	0.704409	0.345976	0.569058	0.715118	0.064734
6	0.860532	0.794088	0.389394	0.624288	0.755295	0.395894
7	0.526868	0.597565	0.028711	0.012798	0.025958	0.030609
8	0.434695	0.702096	0.108255	0.051448	0.000352	0.106071
